# *Addendum*: Qian, C.; Fang, Q.; Wang, L.; Ye, G.Y. Molecular Cloning and Functional Studies of Two Kazal-Type Serine Protease Inhibitors Specifically Expressed by *Nasonia vitripennis* Venom Apparatus. *Toxins* 2015, *7*, 2888–2905

**DOI:** 10.3390/toxins7093636

**Published:** 2015-09-11

**Authors:** Cen Qian, Qi Fang, Lei Wang, Gong-Yin Ye

**Affiliations:** 1 College of Life Science, Anhui Agricultural University, Hefei 230036, China; E-Mails: qiancenqiancen@163.com (C.Q.); wanglei20041225@163.com (L.W.); 2 State Key Laboratory of Rice Biology, Key Laboratory of Agricultural Entomology, Institute of Insect Sciences, Zhejiang University, Hangzhou 310058, China; E-Mail: fangqi@zju.edu.cn

The authors would like to replace the Acknowledgements section with the following:

**“Acknowledgments**
  This research was supported by grants from the National Program on Key Basic Research Projects (973 Program, 2013CB127600), the National Natural Science Foundation of China (Grant Nos. 31272098, 31472038, 31402018), the Research Fund for the Doctoral Program of Higher Education of China (Grant Number 2012010113004), the National Science Fund for Innovative Research Groups of Biological Control (Grant No. 31321063), the Zhejiang Provincial Natural Science Foundation of China (Grant Number Y14C140006) and the Fundamental Research Funds for the Central Universities (Grant Number 2014FZA6014).”

